# Modulation of radiochemoimmunotherapy-induced B16 melanoma cell death by the pan-caspase inhibitor zVAD-fmk induces anti-tumor immunity in a HMGB1-, nucleotide- and T-cell-dependent manner

**DOI:** 10.1038/cddis.2015.129

**Published:** 2015-05-14

**Authors:** N Werthmöller, B Frey, R Wunderlich, R Fietkau, U S Gaipl

**Affiliations:** 1 Department of Radiation Oncology, University Hospital Erlangen, Friedrich-Alexander-Universität Erlangen-Nürnberg, Germany

## Abstract

One prerequisite that radiotherapy (RT) and chemotherapy (CT) result in anti-tumor immune responses is triggering of immunogenic cell death forms such as necroptosis. The latter is inducible by inhibition of apoptosis with the pan-caspase inhibitor zVAD-fmk. The design of multimodal therapies that overcome melanoma's resistance to apoptosis is a big challenge of oncoimmunology. As hints exist that immune stimulation by hyperthermia (HT) augments the efficacy of melanoma therapies and that tumors can be sensitized for RT with zVAD-fmk, we asked whether combinations of RT with dacarbazine (DTIC) and/or HT induce immunogenic melanoma cell death and how this is especially influenced by zVAD-fmk. Necroptosis was inducible in poorly immunogenic B16-F10 melanoma cells and zVAD-fmk generally increased melanoma cell necrosis concomitantly with the release of HMGB1. Supernatants (SNs) of melanoma cells whose cell death was modulated with zVAD-fmk induced an upregulation of the activation markers CD86 and MHCII on macrophages. The same was seen on dendritic cells (DCs), but only when zVAD-fmk was added to multimodal tumor treatments including DTIC. DCs of MyD88 KO mice and DCs incubated with SNs containing apyrase did not increase the expression of these activation markers on their surface. The *in vivo* experiments revealed that zVAD-fmk decreases the tumor growth significantly and results in a significantly reduced tumor infiltration of Tregs when added to multimodal treatment of the tumor with RT, DTIC and HT. Further, a significantly increased DC and CD8+ T-cell infiltration into the tumor and in the draining lymph nodes was induced, as well as an increased expression of IFN*γ* by CD8+ T cells. However, zVAD-fmk did not further reduce tumor growth in MyD88 KO mice, mice treated with apyrase or RAG KO mice. We conclude that HMGB1, nucleotides and CD8+ T cells mediate zVAD-fmk induced anti-melanoma immune reactions in multimodal therapy settings.

The cancer immune editing concept raised by Schreiber and colleagues^1^ and the findings that distinct chemotherapeutic agents induce immunogenic cancer cell death forms^2^ opened our minds that standard tumor therapies alone and especially in combination with further immune therapies are capable of inducing anti-tumor immune responses.^3^ The phenotype of the tumor cells and the tumor microenvironment are altered during therapy and, thereby, the tumor might become visible for the immune system.^4^ A main prerequisite for induction of anti-tumor immunity is triggering of immunogenic tumor cell death forms.^5^

Apoptosis is non- or even anti-inflammatory.^6^ In contrast, necrotic cells bear per se a high inflammatory and immunogenic potential. Damage-associated molecular patterns (DAMPs) are released because the plasma membrane of necrotic cells is disturbed.^7, 8^ Danger signals as the high mobility group protein B1 (HMGB1) and the nucleotide adenosine triphosphate (ATP) activate DCs, foster cross-presentation of antigens and consecutively the activation of T cells.^9^ DAMPs therefore link radio- and/or chemotherapy-induced local alterations of the tumor cells and subsequent systemic anti-tumor immune reactions.^10, 11^ HMGB1 is mostly passively released by therapy-induced necrotic tumor cells.^12^ The activation of DCs by HMGB1 is induced by its binding to TLR2 or TLR4.^13, 14^ HMGB1 is further required for the migration of maturing DCs.^15^ The nucleotide ATP is often actively emitted and acts on purinergic receptors, especially on P2RX7.^16, 17^

Activation of DCs is crucial for the success of multimodal tumor treatments.^18^ Several preclinical and clinical studies have demonstrated that tumor cell death induced by radiochemotherapy in combination with intratumoral DC injection induces strong anti-tumor immune responses in several tumor entities.^19, 20, 21^ These responses can be enhanced by hyperthermia (HT). Mild HT is an additive therapy to radiotherapy (RT) and/or chemotherapy (CT) in which tumor tissue is locally heated to temperatures of 40–44 °C for a time period of 1 h. HT fosters protein aggregation and aggravates radiation- and chemotherapy-induced repair of DNA damage.^22^ In addition, locally applied HT is capable of inducing systemic anti-tumor responses.^23^

Melanoma is the most dangerous form of skin cancer and its response to CT and RT is poor.^24^ To overcome melanoma's resistance to apoptosis, the search for multimodal treatments that aim of inducing immunogenic cell death forms is a big challenge of innovative oncoimmunology,^25^ as much as to understand the mechanisms of therapy-induced immunogenic melanoma cell death. Nowadays, evidence has come up that necrosis as immunogenic cell death form can also occur in a programmed manner.^26, 27^ Necroptosis is independent of caspases and mainly occurs when caspases are not activated or inhibited.^28^ The pan-caspase inhibitor zVAD-fmk has been shown to inhibit apoptosis and concomitantly foster necroptosis.^29^ Further, encouraging preclinical studies have been performed using caspase inhibitors to reduce apoptosis in neurological diseases^30^ and to reduce angiogenesis in solid tumors.^31^ First hints exist that immune stimulation by HT is capable of augmenting the efficacy of CT and RT treatments in melanoma^32^ and that solid tumors can be rendered more sensitive to radiation by treatment with the pan-caspase inhibitor zVAD-fmk.^31^ Meaningful data regarding potential clinical efficacy of caspase inhibitors such as zVAD-fmk will only be yielded if the cell death pathways stimulated in model systems reflect that triggered in patients.^33^ Therefore, we examined here for the first time whether combinations of the clinically relevant single dose of RT of 2 Gy with the only for metastatic melanoma FDA-approved CT agent dacarbazine (DTIC) or combinations with HT (41.5 °C for 1 h) induce immunogenic melanoma cell death and how zVAD-fmk is capable of improving the melanoma's immunogenicity by modulating the therapy-induced melanoma cell death.

## Results

### Single treatments of melanoma cells with clinically relevant dosage of RT, DTIC or HT do not induce apoptosis or necrosis

**Figure 1:**
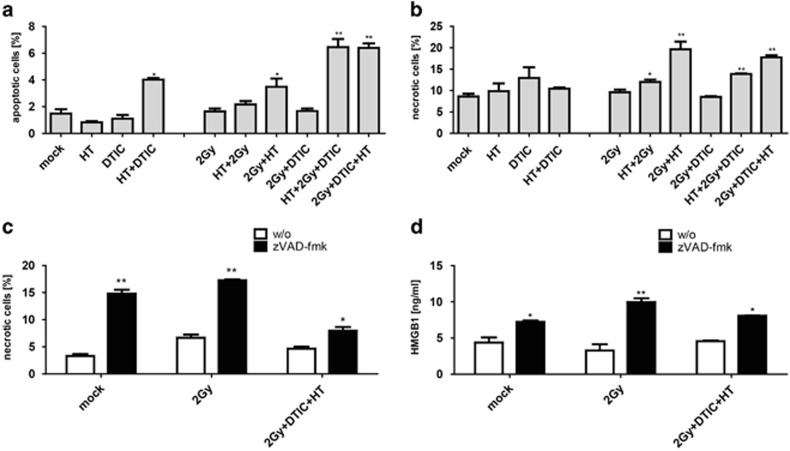
Cell death and release of the danger signal HMGB1 of melanoma cells after single and multimodal treatments with RT, DTIC and HT in the absence or presence of zVAD-fmk. The cell death forms of B16 mouse melanoma cells were analyzed with two color flow cytometry after staining with AnxA5-FITC and PI, 72 h (**a** and **b**) or 24 h (**c**) after the respective treatment. The release of HMGB1 was analyzed 24 h after the treatments with ELISA (**d**). Apoptotic cells are defined as AnxA5+/PI− and necrotic ones as AnxA5+/PI+. Representative data of one out of four experiments, each performed in triplicates, are presented as mean±S.D. *P<0.05; **P<0.01 related to untreated (mock) B16 cells (**a** and **b**) or to samples without (w/o) zVAD-fmk (**c** and **d**). DTIC at a concentration of 250 *μ*M; Gy, Gray; HT with 41.5 °C for 1 h; zVAD-fmk, pan-caspase inhibitor. The order of irradiation with 2 Gy and HT indicated on the *x* axis determines the chronology of the treatment

The response of melanoma cells to ionizing irradiation applied in classical RT and to DTIC is often poor.^24^ First hints exist that HT is capable of augmenting the efficacy of RT and CT treatments also in melanoma.^32, 34^ We observed that single treatments of the melanoma cells with RT, DTIC or HT do not significantly alter the percentage of apoptotic and necrotic cells (Figures 1a and b).

### RT combined with HT enhances melanoma cell apoptosis and necrosis

However, the percentage of apoptotic and necrotic melanoma cells was increased when RT and HT were given in combination, while necrosis dominated over apoptosis (Figures 1a and b). Administration of irradiation before HT (2 Gy+HT) was more efficient in cell death induction as the other way round (HT+2 Gy). Regarding the chemosensitization effect of HT, only apoptosis of melanoma cells was slightly, but significantly increased (Figure 1a). The highest percentage of apoptotic plus necrotic cells was achieved by combining RT with DTIC and HT (Figures 1a and b).

### Necroptosis is inducible in melanoma cells and correlates with release of HMGB1

Blocking apoptosis with the pan-caspase inhibitor zVAD-fmk resulted in increased amounts of necrotic cells. This was independent of the death stimuli (Figure 1c). The addition of the necroptosis inhibitor nec-1 blocked the zVAD-fmk-induced tumor cell necrosis (Supplementary Figure 1A). Further, the mRNA expression of MLKL being as protein a component of the necroptosome was enhanced when zVAD-fmk was present (Supplementary Figure 1B). Fostering melanoma cell necrosis with zVAD-fmk increased the amount of HMGB1 in the SNs of the tumor cells. This increased release of HMGB1 correlated with the percentage of necrotic tumor cells (Figures 1c and d).

### Increased surface expression of MHCII and CD86 on macrophages by SNs of melanoma cells whose cell death was modulated with zVAD-fmk

As inhibition of caspases by zVAD-fmk has been suggested to be a promising strategy to enhance the efficacy of RT in solid tumors ^31^ and because the immune system contributes to radiochemotherapy-induced tumor control,^35^ we next investigated whether SNs of melanoma cells that have been exposed to zVAD-fmk in addition to RT, DTIC, and/or HT impact on activation and function of macrophages and DCs.

**Figure 2:**
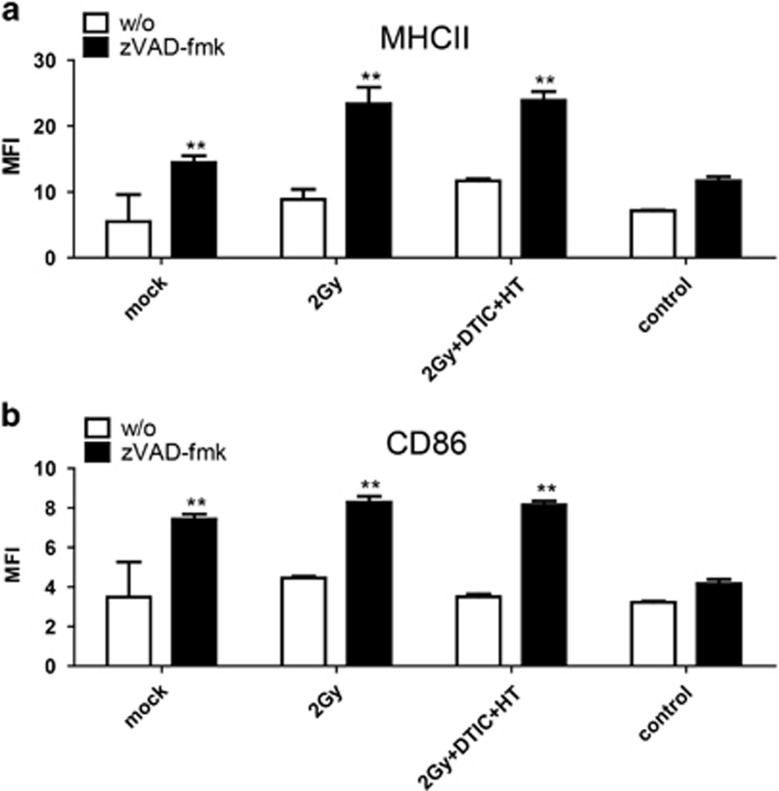
Surface expression of activation markers on macrophages after contact with SNs of treated melanoma cells in the presence or absence of zVAD-fmk. The expression of the activation markers MHCII (**a**) and CD86 (**b**) on the surface of peritoneal mouse macrophages of C57/BL6 mice was analyzed by multicolor flow cytometry after contact with SNs of B16 mouse melanoma cells obtained 24 h after the respective treatments. The tumor cells were treated with ionizing irradiation with 2 Gy alone or in combination with the chemotherapeutic agent DTIC (250 *μ*M) and HT (41.5 °C for 1 h), in each case in the absence (w/o) or presence of the pan-caspase inhibitor zVAD-fmk (50 *μ*M) (**a** and **b**). Representative data of one out of three experiments, each performed in triplicates, are presented as mean±S.D. **P<0.01 related to samples without (w/o) inhibitor; MFI, mean fluorescence intensity; mock, SNs of untreated tumor cells; control, culture medium with or without zVAD-fmk

**Figure 3:**
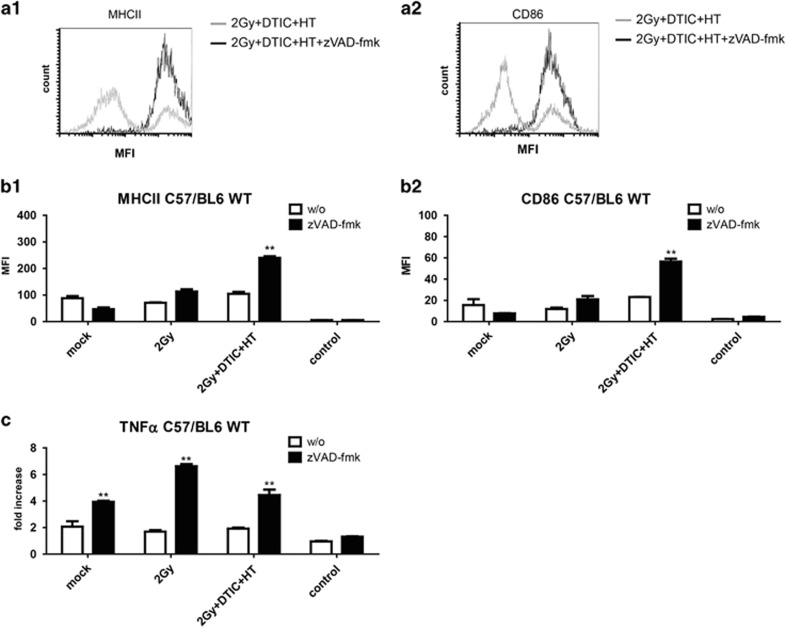
Surface expression of activation markers on DCs and release of TNF*α* by DCs after contact with SNs of treated melanoma cells in the presence or absence of zVAD-fmk. The expression of the activation markers MHCII (**a1** and **b1**) and CD86 (**a2** and **b2**) on the surface of bone marrow-derived DCs of C57/BL6 wild-type mice was analyzed by multicolor flow cytometry after contact with SNs of B16 mouse melanoma cells obtained 24 h after the respective treatments. The tumor cells were treated with ionizing irradiation with 2 Gy alone or in combination with the chemotherapeutic agent DTIC (250 *μ*M) and HT (41.5 °C for 1 h), in each case in the absence (w/o) or presence of the pan-caspase inhibitor zVAD-fmk (50 *μ*M). In **a1** and **a2**, representative histograms of the expression of MHCII or CD86 are displayed after contact of the DCs with SNs of melanoma cells that had been treated with RT, DTIC and HT in the absence (grey) or presence of zVAD-fmk (black). The SNs of DCs after contact with the SNs of the melanoma cells were analyzed for TNF*α* by ELISA (**c**). Data of three experiments, each performed in triplicates, are presented as mean±S.D. **P<0.01 related to samples without (w/o) inhibitor; MFI, mean fluorescence intensity; mock, SNs of untreated tumor cells; control, culture medium with or without zVAD-fmk

SNs of neither mock-treated melanoma cells nor of treated ones altered the expression of the activation markers MHCII and CD86 on macrophages. However, the modulation of melanoma cell death by zVAD-fmk resulted in tumor cell SNs that induced a significant increased expression of MHCII and CD86 on the surface of macrophages, independent of the death stimulus (Figure 2). To prove that this is not a direct effect of zVAD-fmk on the immune cells, zVAD-fmk alone was added to macrophages or DCs. No influence on the expression of activation markers was observed neither for macrophages (Figure 2) nor for DCs (Figures 3b1 and b2).

### Increased surface expression of MHCII and CD86 on DCs by SNs of melanoma cells whose cell death was induced by combination of RT, DTIC and HT and modulated with zVAD-fmk

**Figure 4:**
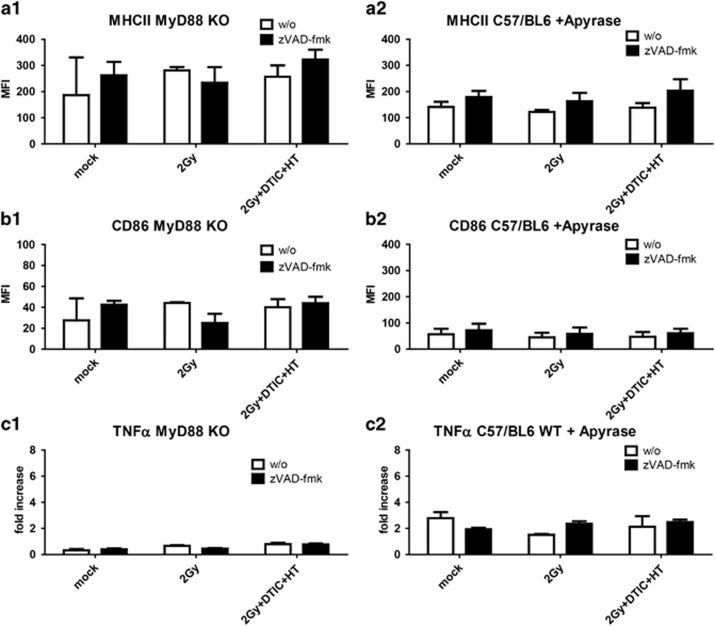
Impact of TLR signaling and nucleotides on the surface expression of activation markers on DCs and release of TNF*α* by DCs after contact with SNs of treated melanoma cells in the presence or absence of zVAD-fmk. The expression of the activation markers MHCII (**a1** and **a2**) and CD86 (**b1** and **b2**) on the surface of bone marrow-derived DCs of MyD88 KO mice (**a1** and **b1**) or DCs of C57/BL6 wild-type mice in the presence of Apyrase (**a2** and **b2**) was analyzed by multicolor flow cytometry, after contact with SNs of B16 mouse melanoma cells obtained 24 h after the respective treatments. The tumor cells were treated with ionizing irradiation with 2 Gy alone or in combination with the chemotherapeutic agent DTIC (250 *μ*M) and HT (41.5 °C for 1 h), in each case in the absence (w/o) or presence of the pan-caspase inhibitor zVAD-fmk (50 *μ*M). The SNs of DCs after contact with the SNs of the melanoma cells were analyzed for TNF*α* by ELISA (**c1** and **c2**). Data of three experiments, each performed in triplicates, are presented as mean±S.D. **P<0.01 related to samples without (w/o) inhibitor; MFI, mean fluorescence intensity; mock, SNs of untreated tumor cells

In contrast to macrophages, the expression of MHCII and CD86 on the surface of DCs could not generally be increased by SNs of melanoma cells whose cell death was modulated by zVAD-fmk. However, cell death induction with DTIC in multimodal application with RT and HT together with zVAD-fmk led to significant increased expression of MHCII and CD86 on DCs (Figures 3a and b). This was in part dependent on HMGB1, because no upregulation of the activation markers was observed when using DCs of MyD88 KO mice (Figures 4a1 and b1) or when incubating the tumor cell SNs with anti-HMGB1 antibody, respectively (Supplementary Figure 2). Further nucleotides impact on it, as incubation of the SNs with Apyrase also impaired the expression of these activation markers on DCs (Figures 4a2 and b2).

### SNs of melanoma cells whose cell death was modulated with zVAD-fmk induce an increased secretion of TNF*α* by DCs

Contact of DCs with SNs of melanoma cells whose cell death was modulated by zVAD-fmk resulted in an increased TNF*α* secretion (Figure 3c). This was independent of the treatment just as the HMGB1 release (Figure 1d). The secretion of TNF*α* was dependent on HMGB1 and on nucleotides, because using DCs of MyD88 KO mice or incubating B16-F10 cell SNs with Apyrase did not alter the release of TNF*α* by DCs (Figures 4c1 and c2). Incubation of macrophages or DCs with SNs of treated melanoma cells did not impact on the phagocytosis of the dying melanoma cells by these immune cells. Further, the migration of macrophages or DCs towards the melanoma cells was also not influenced by this (data not shown).

### Multimodal tumor treatment with RT, DTIC and HT in combination with zVAD-fmk results in long-lasting tumor growth retardation in dependence of HMGB1 and nucleotides

**Figure 5:**
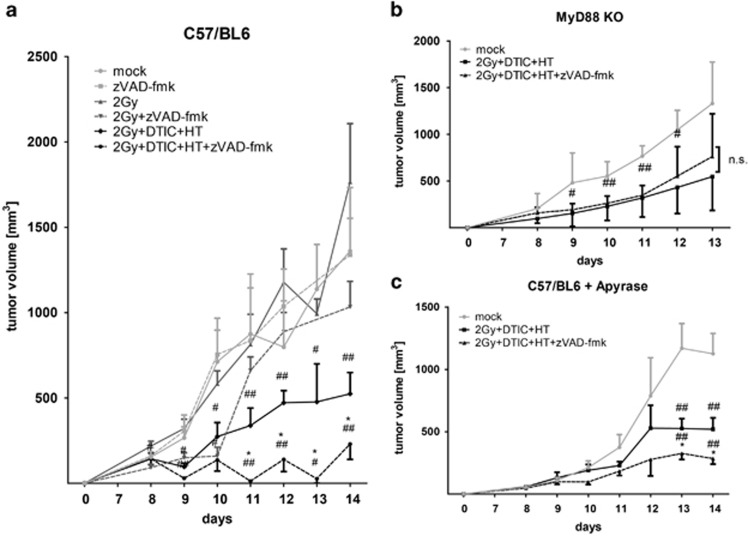
*In vivo* growth of B16 tumors after fractionated RT, DTIC and HT in the absence or presence of zVAD-fmk and the impact of TLR signaling and nucleotides on it. The tumor growth of syngeneic B16 tumors in wild-type C57/BL6 (**a**), MyD88 KO (**b**) or wild-type C57/BL6 mice after systemic treatment with Apyrase (**c**) is displayed. The tumors were locally irradiated at days 8, 9 and 10 with a clinically relevant single dose of 2 Gy using a linear accelerator. Two hours after the irradiation, DTIC (2 mg/mouse at day 8 and 10) and zVAD-fmk (2 mg/kg at day 8, 9 and 10) were injected i.p. Apyrase (**c**) was injected i.v. 1 h after irradiation (25 U/mouse at day 8). HT was performed 4 h after irradiation at day 8 and 10. For this, the mice were anesthetized and the tumors were heated locally under temperature control to 41.5 °C for 30 min using the BSD50 hyperthermia system. The tumor volume was monitored with an electronic caliper. Joint data of three independent experiments, each with three mice per group, are presented as mean±S.D. ^#^*P*<0.05; ^##^*P*<0.01 related to untreated tumors (mock); *P<0.05 related to tumors treated with RT, DTIC and HT in the absence of zVAD-fmk; n.s., not significant

Treatment of B16 tumor-bearing mice with fractionated RT with a single dose of 2 Gy did not significantly delay tumor growth. The same was true for single treatment with zVAD-fmk or its combination with fractionated RT (Figure 5a). However, combination of fractionated RT with DTIC and HT resulted in significant reduction of the tumor volume. The most pronounced and long-lasting tumor growth inhibition was observed when zVAD-fmk was added to the multimodal treatment with fractionated RT, DTIC and HT (Figure 5a). Significant tumor growth retardation by combination of fractionated RT, DTIC and HT was also observed in MyD88 KO mice (Figure 5b) and when injecting Apyrase simultaneously in tumor-bearing wild-type mice (Figure 5c). However, the addition of zVAD-fmk did not further reduce the tumor volume in MyD88KO mice as observed in the wild-type situation (Figures 5b and a) and it was less effective when Apyrase was simultaneously injected in wild-type mice (Figures 5c and a).

### zVAD-fmk impacts on immune cell infiltration into the tumor

**Figure 6:**
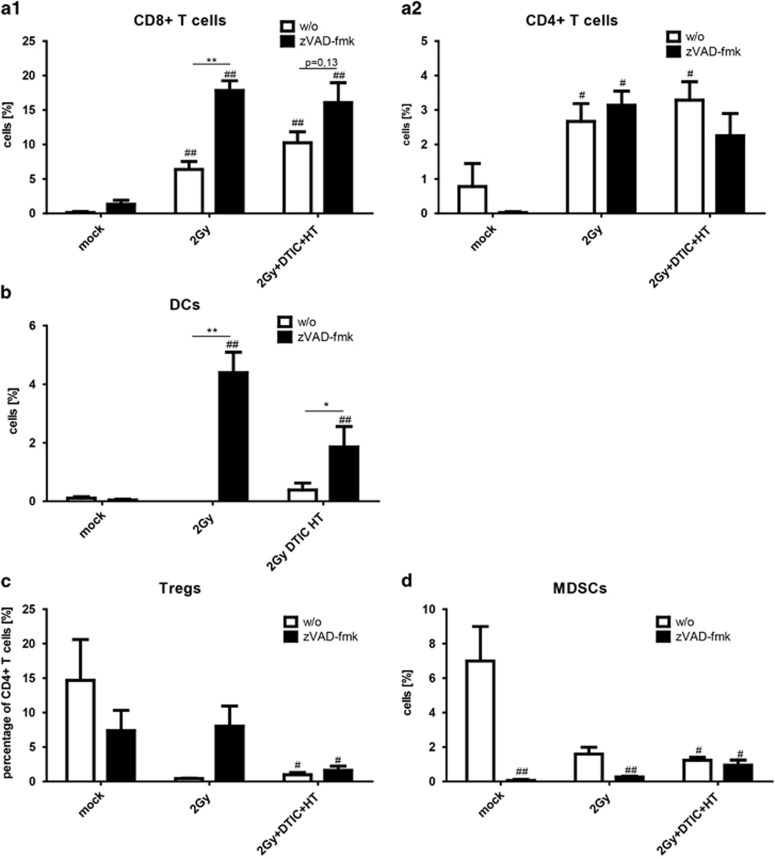
Immune cell infiltration into B16 tumors in C57/BL6 mice after fractionated RT, DTIC and HT in the absence or presence of zVAD-fmk. The infiltration of immune cells (CD8+ T cells (**a1**), CD4+ T cells (**a2**), DCs (**b**), Tregs (**c**) and MDSCs (**d**)) was analyzed by multicolor flow cytometry, 24 h after the last treatment of the tumors. The latter were before locally irradiated at days 8, 9 and 10 with a clinically relevant single dose of 2 Gy using a linear accelerator. Two hours after the irradiation, DTIC (2 mg/mouse at day 8 and 10) and zVAD-fmk (2 mg/kg at day 8, 9 and 10) were injected i.p. HT (41.5 °C for 30 min) was performed 4 h after irradiation at day 8 and 10. Joint data of three independent experiments, each with two mice per group, are presented as mean±S.D. ^#^*P*<0.05; ^##^*P*<0.01 related to untreated tumors (mock).*P<0.05, **P<0.01 related to tumors treated with RT, DTIC and HT in the absence of zVAD-fmk

A significant increased infiltration of both CD8+ and CD4+ T cells into the tumor was observed after fractionated RT when combined with DTIC and HT. The addition of zVAD-fmk further increased the percentage of infiltrating CD8+ T cells (Figure 6a1), but not of CD4+ T cells (Figure 6a2). The infiltration of DCs into the tumor was only enhanced by combination of RT with zVAD-fmk (Figure 6b). The infiltration of Tregs was significantly decreased after combined treatment both in the presence or absence of zVAD-fmk. The percentage of tumor-infiltrating MDSCs was decreased by all examined treatments (Figure 6d).

### Multimodal tumor treatment with RT, DTIC and HT in combination with zVAD-fmk increases infiltration of DCs and of CD8+ T cells into lymph nodes and the IFN*γ* expression by CD8+ T cells

**Figure 7:**
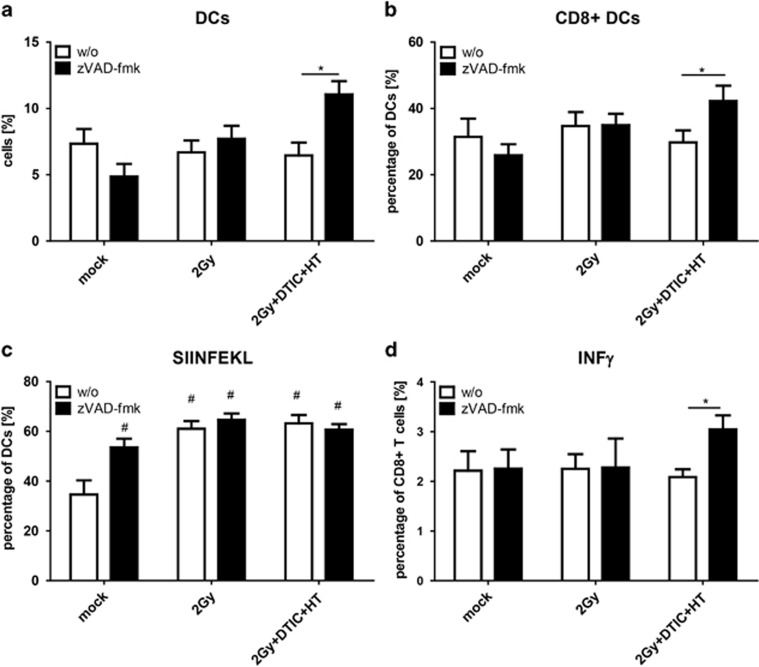
Infiltration of DCs into lymph nodes of B16-OVA tumor-bearing OT1 mice after fractionated RT, DTIC and HT in the absence or presence of zVAD-fmk and related CD8+ T-cell activation. The infiltration of DCs (**a**) and especially that of CD8+ DCs (**b**) into the draining lymph nodes of OT1 mice was analyzed by multicolor flow cytometry, 96 h after the last treatment of the B16-OVA tumor according to the treatments already indicated in Figures 5 and 6. Both presentation of SIINFEKL by DCs (**c**) and percentage CD8+ T cells expressing IFN*γ* after *ex vivo* re-stimulation with OVA peptide were analyzed by multicolor flow cytometry, 7 days after the indicated treatment. Joint data of three independent experiments, each with two mice per group, are presented as mean±S.D. ^#^*P*<0.05 related to untreated tumors (mock); *P<0.05 related to tumors treated with RT, DTIC and HT in the absence of zVAD-fmk

For an effective immune response against the tumor not only the number, but also the phenotype including the activation status of the immune cells are crucial. Only multimodal tumor treatment with RT, DTIC and HT in combination with zVAD-fmk increased the percentage of DCs in lymph nodes (Figure 7a), and especially that of CD8+ DCs (Figure 7b). The latter are specialized for cross-presentation of antigens.^36^ Nevertheless, the presentation of a tumor model antigen was as effective in the presence or absence of zVAD-fmk. All treatments enhanced the cross-presentation of OVA257-264; only DCs of mock-treated mice presented less model tumor antigen (Figure 7c). However, only T cells of mice whose tumor was treated with RT, DTIC and HT in combination with zVAD-fmk expressed significant higher amounts of IFN*γ* after re-stimulation (Figure 7d).

### Multimodal tumor treatment with RT, DTIC and HT in combination with zVAD-fmk retards tumor growth in a T-cell-dependent manner

**Figure 8:**
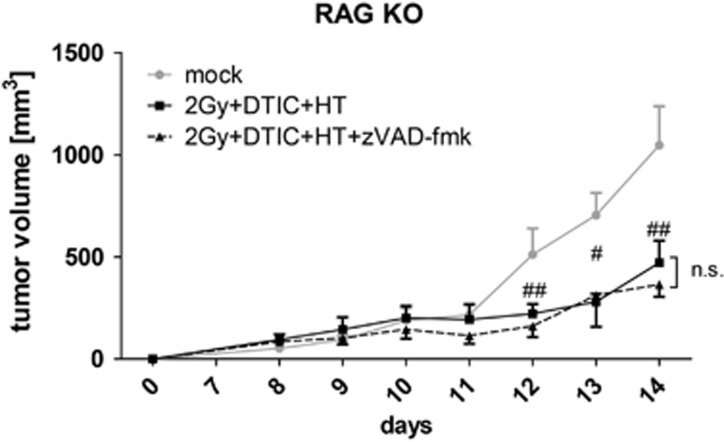
*In vivo* growth of B16 tumors in RAG KO mice after fractionated RT, DTIC and HT in the absence or presence of zVAD-fmk. The growth of syngeneic B16 tumors in RAG KO mice is displayed. The tumors were either left untreated (mock) or were locally irradiated at days 8, 9 and 10 with a clinically relevant single dose of 2 Gy using a linear accelerator. Two hours after the irradiation, DTIC (2 mg/mouse at day 8 and 10) and zVAD-fmk (2 mg/kg at day 8, 9 and 10) were injected i.p. Joint data of three independent experiments, each with two mice per group, are presented as mean±S.D. ^##^*P*<0.01 related to untreated tumors (mock). n.s., not significant

The enhanced infiltration and activation of CD8+ T cells after multimodal tumor treatment and cell death modulation with zVAD-fmk had given hints that the observed tumor growth retardation by zVAD-fmk is dependent on T cells. To prove this, *in vivo* experiments were performed with B16-F10 tumor-bearing RAG-2-deficient (RAG KO) mice. Here, zVAD-fmk in combination with RT, DTIC and HT did not further decrease the tumor growth significantly in comparison with the multimodal treatment alone (Figure 8).

## Discussion

Under certain clinical conditions, RT should be considered as treatment for melanoma.^37^ HT as an adjuvant has been clinically proven to improve local control of malignant melanoma.^38^ Its chemoresistance can be overcome in a tumor cell-selective manner by interfering with anti-apoptotic Bcl-2 family members.^39, 40^ Modulation of apoptotic cell death is therefore a promising strategy to combat cancer and inflammatory diseases in general. Here, caspases represent key targets for drug development as they are central in initiation and execution of cell death and in maturation of inflammatory cytokines. Pan-caspase inhibitors such as zVAD-fmk and IDN-6556 (F-03491390) have been proven in preclinical and currently running phase I/II clinical trials to be suitable drugs with manageable side effects.^41, 42^

Besides local tumor control, systemic anti-tumor effects should be induced by modulation of cell death pathways.^3, 4, 43^ Although zVAD-fmk enhanced B16 necrosis (Figure 1c), it did not impact on the clonogenic potential of the tumor cells (Supplementary Figure 3A). However, a slight, but significant reduction of tumor cell proliferation induced by zVAD-fmk alone was observed (Supplementary Figure 3B). This suggests that besides its contribution to systemic immune activation against the tumor, zVAD-fmk also might locally act on the tumor. However, no further reduction of tumor growth by zVAD-fmk was observed when being combined with RT (Supplementary Figure 3).

Multimodal treatment with RT, DTIC and HT resulted in the highest percentages of both apoptotic and necrotic melanoma cells. Especially a mixture of apoptosis and necrosis is supposed to possess high immunogenic potential,^10^ as also proven for melanoma vaccines consisting of lethally irradiated melanoma cells.^44^ Apoptotic cells release chemotactic factors to attract immune cells,^45^ and the release of DAMPs by necrotic cells then activates them. As the pan-caspase inhibitor zVAD-fmk has been shown to be capable of inducing immunogenic necrotic cell death forms in selected tumor entities such as fibrosarcoma^46^ and to better understand the molecular mechanisms of therapy-induced melanoma cell death, we were interested whether necroptosis is inducible in B16-F10 cells. Indeed, blocking B16 apoptosis with zVAD-fmk resulted in increased necrotic cell death. The induction of B16 necrosis by zVAD-fmk could be blocked by the necroptosis inhibitor, nec-1 (Supplementary Figure 1A), and addition of zVAD-fmk to irradiated B16 cells increased the mRNA level of MLKL (Supplementary Figure 1B).^47^

Also independent of the death stimuli, zVAD-fmk induced the release of HMGB1 by B16 cells (Figure 1d). Danger signals in general enhance maturation and antigen presentation of macrophages and DCs. These innate immune cells are the first ones that are recruited to the tumor. They regulate the cells of the adaptive immune system and are therefore crucial for success of an immune-based tumor therapy.^48^ The amount of released HMGB1 impacted especially on the increased expression of MHCII and CD86 on the surface on macrophages (Figure 2). In the case of DCs, the release of TNF*α* also correlated with it (Figure 3c). However, the expression of MHCII and CD86 on the surface on DCs was only increased after contact with SNs of B16 cells that had been treated with RT, DTIC, HT and zVAD-fmk. This indicates that, here, not only the amount of HMGB1 is crucial, but most likely also its redox state and further danger signals.^49^ A reductive environment may be important to maintain the bioactivity of HMGB1. *In vivo*, again, only combination of the multimodal treatment with zVAD-fmk and not only RT with zVAD-fmk resulted in highly significant tumor growth retardation (Figure 5). This suggests that other danger signals such as nucleotides in addition to HMGB1 are responsible for the therapy-induced immune activation (Figures 4a2 and b2 and Figure 5c). Further, the redox state of HMGB1 and not only the amount might also impact on it, because not only nucleotides are the reason for therapy-induced tumor growth retardation; even in the presence of apyrase, multimodal treatment with zVAD-fmk significantly further retards tumor growth when compared with multimodal treatment only (Figure 5c). These results indicate that multiple mechanisms are responsible for induction of anti-tumor immunity by zVAD-fmk when added to multimodal treatments. While early activation markers such as CD86 were increased on the surface of DCs, SNs of treated B16 cells did not impact on the expression of CD80 or CD40 on DCs (Supplementary Figure 4).^50, 51, 52^

As the upregulation of distinct activation markers was no longer observed on DCs of MyD88 KO mice (Figures 4a1 and b1) or SNs of tumor cells that had been incubated with Apyrase (Figures 4a2 and b2), we conclude that a combination of released HMGB1 and nucleotides like ATP is responsible for the activation of DCs. To confirm the impact of HMGB1 on activation of DCs, SNs of the treated tumor cells were supplemented with a neutralizing antibody against HMGB1 and added to the DCs afterwards. As shown in the Supplementary Figure 2, the increased expression of the activation markers on DCs was dependent on HMGB1. The latter activates immune cells via binding to the receptor for advanced glycation end products (RAGE) or especially to Toll-like receptor (TLR) 2 and TLR4. MyD88 KO mice have defects in TLR signaling and our results show that zVAD-fmk did not further retard growth of B16 tumors treated with RT, DTIC and HT in these mice (Figure 5b), again indicating that HMGB1 *in vivo* is a key player in zVAD-fmk-induced anti-tumor immune responses.

Apyrase is a calcium-activated plasma membrane-bound ATP diphosphohydrolase that catalyzes the hydrolysis of nucleotides like ATP to AMP and inorganic phosphate.^53^ Removing extracellular nucleotides with apyrase treatment has been shown not only to prevent IL-1*β* accumulation, but also the production of inflammasome-independent cytokines such as TNF.^54^ The secretion of TNF*α* by DCs after contact with SN of B16 tumor cells that have been treated with zVAD-fmk was also dependent on both nucleotides and TLR signaling (Figures 4c2 and c1). As the half-life of zVAD-fmk is short (<40 min),^55^ a direct effect of the pan-caspase inhibitor on the immune cells is further excluded.

The *in vivo* studies demonstrated that combination of RT, DTIC and HT with zVAD-fmk induced the strongest tumor growth retardation in a HMGB1- and nucleotide-dependent manner (Figure 5). Fractionated irradiation alone did not affect the tumor growth, reflecting the radioresistance of melanoma also in our model system. While the triple treatment with RT, DTIC and HT in the absence of zVAD-fmk already induced a significant increased infiltration of CD8+ T cells into the tumor, zVAD-fmk in combination with RT further enhanced this (Figure 6a1). The infiltration of DCs into the tumor was only significantly enhanced when zVAD-fmk was given in combination with fractionated RT (Figure 6b). Tregs and MDSCs that exert immune suppression in melanoma^56^ were significantly reduced in the tumor, both after triple treatment in the presence or absence of zVAD-fmk (Figures 6c and d).

Of note is that only combination of RT, DTIC and HT with zVAD-fmk-induced increased amounts of DCs, and especially of CD8+ DCs which are specialized to cross-present antigen^57^ in the lymph nodes of OT1 mice (Figures 7a and b). However, zVAD-fmk in general fostered the presentation of the model antigen OVA257-264 by DCs, as it was also the case when treating the tumor with RT in single or multimodal settings (Figure 7c). However, the activation of CD8+ T cells, monitored by increased expression of IFN*γ* after restimulation with OVA peptide, was only observed after treatment of the tumor with RT, DTIC and HT in the presence of zVAD-fmk (Figure 7d).

That T cells are involved in immune reactions against B16 tumors treated with RT, DTIC and HT in combination with zVAD-fmk was further underpinned by performing the *in vivo* experiments with RAG KO mice that lack functional B and T cells. No significant further tumor growth retardation was observed when zVAD-fmk was combined with the multimodal treatment (Figure 8).

To summarize, zVAD-fmk induced immunogenic melanoma cell necroptosis *in vitro* and tumor growth retardation *in vivo* when combined with fractionated RT, DTIC and HT. This was dependent on HMGB1 and nucleotides. Activation of CD8+ T cells by DCs seems to be one key mechanism of zVAD-fmk-triggered anti-melanoma immune responses. Moretti *et al.*^31^ already demonstrated that zVAD-fmk acts as radiosensitizer for lung and breast cancer cells. It significantly further retarded the growth of xenogeneic tumors treated with fractionated RT (5 × 2 Gy) and increased the expression of HMGB1 as observed in tumor tissue sections. Distinct irradiation schemes, chemotherapeutic drugs, HT and cell death-modifying agents such as zVAD-fmk bear high potential as immune-modulating agents besides their direct cytotoxic effects on the tumor cells. The abscopal effect, a phenomenon in which local RT in combination with further immune modulation^58^ is associated with the regression of cancer at a distance from the irradiated site,^4^ raises the hope that multimodal treatments that induce an activation of immune cells will increase the survival of melanoma patients in the future. Such abscopal effects have already been clinically observed in patients with metastatic lung cancer and melanoma treated with ipilimumab and RT.^59, 60^ Our data suggest including zVAD-fmk in radiochemoimmunotherapy treatment protocols of melanoma to foster the induction of immunogenic melanoma cell death and concomitantly T-cell-mediated anti-melanoma immunity.

## Materials and Methods

### Cell culture

The mouse melanoma cell lines B16-F10 and B16-OVA (ATCC, Manassas, VA, USA) both derived from C57/BL6 mice were cultured in RPMI 1640 medium with stable glutamine (Biochrom, Berlin, Germany), supplemented with 10% heat-inactivated fetal bovine serum (Biochrom), 100 U/ml penicillin and 100 *μ*g/ml streptomycin (Gibco, Carlsbad, CA, USA). The cells were tested negatively for mycoplasma contamination and maintained in 5% CO_2_ atmosphere at 37 °C and 95% relative humidity. The cells were used when they reached 90% confluence.

### Treatment of the melanoma cells

The tumor cells were irradiated with a X-ray generator (120 kV, 22.7 mA, variable time; GE Inspection Technologies, Hürth, Germany) with a single dose of 2 Gy, representing a clinically relevant single dose in RT of tumors. The routinely applied and only FDA-approved chemotherapy drug for metastatic melanoma DTIC (Sigma-Aldrich, Munich, Germany) was used in the concentration of 250 *μ*M and added in parallel to the irradiation of the tumor cells. The pan-caspase inhibitor carbobenzoxy-valyl-alanyl-aspartyl-[Omethyl]-fluoromethyl-ketone (zVAD-fmk, Bachem, Weil am Rhein, Germany) was used at a concentration of 50 *μ*M and the necroptosis inhibitor necrostatin-1 (nec-1) at a concentration of 10*μ*M. Both inhibitors were solved in dimethyl sulfoxide and added directly after the irradiation. For HT, the melanoma cells were treated in a homemade device placed in a cell incubator as described previously.^61^ The variations of the temperature during the treatment were less than 0.2 °C. The cells remained at stable 41.5 °C for 1 h. For combined applications, the tumor cells were stored at 37 °C for 4 h between RT and/or CT and HT treatment, as it is the maximal time frame between the treatments in clinical application. For degradation of extracellular ATP and nucleotides in general, the cells were incubated with 10 U/ml medium Apyrase right after HT.

### Analyses of cell death forms

Cell death and forms were determined by AnnexinA5 (Anx5)-fluorescein isothiocyanate (FITC) and propidium iodide (PI) staining. For analysis of cell death, 1 × 10^5^ cells were transferred in 400 *μ*l of Ringer's solution (B. Braun, Melsungen, Germany) containing 0.2 *μ*g AnxA5-FITC and 0.4 *μ*g PI. After 30 min of incubation at 4 °C in the dark, the samples were analyzed by flow cytometry. AnxA5 protein was expressed and produced in 293 human embryonic kidney cells (FreeStyle 293 Expression System, Life Technologies, Regensburg, Germany) and purified (Life Technologies, GENEART). Labelling with FITC was performed with the FluoroTag FITC Conjugation Kit (Sigma Aldrich, St. Louis, MO, USA) according to the manufacturer's instructions. Double-negative cells were defined as viable, AnxA5^+^/PI^−^ as apoptotic, and AnxA5^+^/PI^+^ as necrotic ones.

### Clonogenic assay and cell growth

The effect of zVAD-fmk on the radiosensitivity of B16-F10 melanoma cells was assessed in a clonogenic assay. Shortly, cells were plated in triplicates in 60 mm dishes (Nunc Thermo Fisher, Waltham, MA, USA) at concentrations estimated to yield 40–150 colonies per dish. The cells were irradiated with 1, 2, 4, 6, 8 or 10 Gy 24 h after seeding. Directly after irradiation, zVAD-fmk (50 *μ*M) was added. After incubation for ~2 weeks, cells were fixed with methylene blue (Sigma-Aldrich, Munich, Germany) for 30 min. Colonies with >50 cells were scored. The growth of the melanoma cells in the presence or absence of zVAD-fmk was monitored by counting the cells with a Neubauer chamber.

### Quantitative real-time PCR for analysis of MLKL expression

RNA was isolated from irradiated melanoma cells by using peqGOLD TriFast (Peqlab, Erlangen, Germany). cDNA was generated from the RNA by using QuantiTect reverse transcription kit (Qiagen, Venlo, the Netherlands). The expression levels of mixed lineage kinase domain-like (MLKL) mRNA were measured by real-time RT-PCR. For the qPCR experiments, SsoFast EvaGreen Supermix (Bio-Rad, München, Germany) and the Bio-Rad CFX Real-Time System were used. The used primer sequences are forward: 5′- TAGCCGGAGGCTACCAAGTAAAGC-3′, reverse:5′-TGTCCGGCTGATGGAATTCTGTG-3′. mRNA levels were normalized to actin.

### Isolation of peritoneal macrophages

Peritoneal macrophages were isolated from C57/BL6 mice. For this purpose, 2.5 ml of 4% (w/v) of Brewer's thioglycollate broth was injected into the peritoneal cavity as described previously by Schleicher *et al.*^62^ Four days after the injection, the peritoneum was washed with 10 ml PBS (Gibco) and the macrophages were cultured in RPMI 1640 medium with stable glutamine (Biochrom), supplemented with 10% heat-inactivated fetal bovine serum (Biochrom), 100 U/ml penicillin and 100 *μ*g/ml streptomycin (Gibco) and characterized by flow cytometry with preceding F4/80 staining.

### Generation of bone marrow-derived DCs

Generation of bone marrow-derived DCs was performed according to the protocol of Lutz *et al.*^63^ Shortly, bone marrow cells were isolated from femur and tibiae of 8–10-week-old C57/BL6 mice by flushing the cleaned bones with RPMI 1640 medium. The bone marrow cells were then cultured in DC medium (RPMI 1640) medium containing 10% heat-inactivated (30 min at 56 °C) FBS, 100 U/ml penicillin, 100 *μ*g/ml streptomycin, 0.1% *β*-mercaptoethanol (50 mM) and freshly added 200 U/ml mouse GM-CSF. Cells (2 × 10^6^) suspended in 10 ml DC medium were seeded in 100 mm bacteriological petri dishes. At day 3, 10 ml of fresh DC medium were added. At day 6, half of the SN was collected and centrifuged (350 × *g*, 5 min, room temperature). Thereafter, the cell pellet was re-suspended in 10 ml fresh DC medium and returned to the plate. The DCs were harvested at day 8 and used for the activation analyses.

### Analyses of expression of activation markers on macrophages and DCs

Isolated peritoneal macrophages or bone marrow-derived DCs (5 × 10^5^) were co-incubated in 6-well suspension cell plates for 16 h with 1 ml of SNs obtained from B16-F10 tumor cells, 24 h after the respective single or multimodal treatments. Afterwards, the cells were solved with accutase and, to avoid unspecific binding of the staining antibodies to Fc-receptors, incubated for 10 min at 4 °C with Fc-blocking reagent (eBioscience, Frankfurt, Germany). The immune cells were then stained for 30 min at 4 °C with the following fluorescence-labeled antibodies: MHCII-e450 (eBioscience), CD80-PE (BD Pharmingen, New York City, NY, USA), CD86-Alexa Fluor700 (BD Pharmingen) and CD40-APC (eBioscience). Incubation of the cells with just medium, medium with DTIC or zVAD-fmk served as controls. The hereby detected mean fluorescence intensity values after antibody staining were substracted from those resulting after contact of the immune cells with SNs of untreated or treated B16-F10 tumor cells.

Analyses by multicolor flow cytometry were performed with a Gallios Flow Cytometer (Beckman Coulter Inc., Krefeld, Germany).

### Detection of secreted TNF*α*

TNF*α* that was secreted by activated macrophages or DCs was analyzed with a specific enzyme-linked immunosorbent assay (ELISA) kit (Biolegend, San Diego, USA) according to the manufacturer's instructions. For this, SNs of DCs or macrophages were collected 16 h after coincubation with SNs of the melanoma cells.

### Detection of the danger signal HMGB1

The detection of of HMGB1 in SNs of the tumor cells was performed with the ELISA Kit II (Shino-Test Corporation, Tokyo, Japan) according to the manufacturer's instructions. Anti-HMGB1 antibody (Lifespan Biosciences, Nottingham, United Kingdom ) was used to block HMGB1 in the tumor cell SNs for selected experiments.

### Induction of B16 melanomas in C57/BL6, MyD88 KO or RAG KO mice

Eight-week-old female C57/BL6, MyD88 KO or RAG KO mice were used for the *in vivo* experiments. B16-F10 cells (10^6^), solved in 200 *μ*l Ringer's solution, were injected at day 0 into the right shaved flank of the mice. The tumor volumes were monitored over the days. For this, width and length were measured using a digital caliper and tumor volume was calculated according to the following formula: volume (mm^3^)=0.5 × width^2^ (mm^2^) × length (mm).^64^

### Treatment of B16 melanoma-bearing mice with ionizing irradiation, DTIC, HT, zVAD-fmk and Apyrase

At day 8, 9 and 10 after tumor induction, RT was performed. To irradiate the tumor-bearing mice, we manufactured a Plexiglas box which allows the irradiation of three mice at once. The mice were anesthetized before placing them into the box. For the irradiation procedure, the mice were kept under Isoflurane anesthesia to avoid moving of the mice. The tumors were locally irradiated at the indicated days with a clinically relevant single dose of 2 Gy using a linear accelerator (PRIMART, Siemens, Munich, Germany). The planning of the irradiation was conducted using a computer tomography image of the irradiation box and tumor-bearing mice with Philips pinnacle software (Best, Netherlands) to obtain an optimal target volume. To further protect normal tissue, the gantry of the 6 MV linear accelerator was drifted to 340 degree. Two hours after the irradiation DTIC (2 mg/mouse at day 8 and 10) and zVAD-fmk (2 mg/kg at day 8, 9 and 10) were injected i.p. Apyrase was injected i.v. 1 h after irradiation (25 U/mouse at day 8). HT was performed 4 h after irradiation at day 8 and 10. For this, the mice were anesthetized and the tumors were heated locally under temperature control to 41.5 °C for 30 min using the BSD50 hyperthermia system.

All mice were bred under sterile atmosphere at the animal facility of the Friedrich-Alexander-Universität Erlangen-Nürnberg (Franz-Penzoldt-Center). The animal procedures have been approved by the ‘Regierung of Mittelfranken' and were conducted in accordance with the guidelines of Federation of European Laboratory Animal Science Associations (FELASA).

### Analyses of immune cell infiltration by flow cytometry

For analyses of immune cell infiltration, the tumors were processed with a tumor dissociation kit (Miltenyi Biotec, Bergisch Gladbach, Germany). Afterwards, the cells were centrifuged with easycoll separating solution (Biochrom) to discard dead cells. The cells were then stained for 30 min at 4 °C with the following fluorescence labeled antibodies: CD4-PCC5.5 (BD Pharmingen), CD8-PE (BD Pharmingen), CD3-V450 (BD Pharmingen), CD11c-PE (BD Pharmingen) and CD45.2-PCC5.5 (eBioscience). Determination of Tregs was performed with FoxP3 Staining Buffer Set and the antibodies CD4-Vioblue, CD25-AF488 and FoxP3-APC (Miltenyi Biotec). Multicolor flow cytometry was performed with the Gallios Flow Cytometer (Beckman Coulter Inc.).

### Analysis of specific T-cell activation by using OT1 mice and B16-OVA melanoma cells

B16-Ova cells (10^6^) solved in 200 *μ*l Ringer's solution were injected into the right shaved flank of female OT1 mice at day 0. The tumors were treated as described above for the other mice. At day 14, the draining lymph nodes (axillary and inguinal) were removed and single cell suspensions were prepared with cell strainers (pore size of 70 *μ*m). For detection of DCs present in the lymph nodes and presentation of the model tumor antigen, the cells were stained with CD11c-FITC (BD Pharmingen), CD8-APC (BD Pharmingen), OVA257-264 (SIINFEKL)-PE (eBioscience) and CD3-V450 (BD Pharmingen). T-cell activation was measured by analyzing intracellular IFN*γ* after re-stimulation of the cells. For this, 2 × 10^6^ cells were re-stimulated with OVA peptide (10^-7^M) and Golgi Plug for 5 h. Surface staining was performed with CD3-V450 (BD Pharmingen) and CD8-PE (BD Pharmingen) by incubating for 30 min at 4 °C. After washing, the cells were made permeable by addition of Cytofix/Cytoperm and incubation for 20 min at 4 °C. For intracellular staining, the antibody IFN*γ*-Pe-Cy7 (BD Pharmingen) was added and the cells were incubated for another 30 min at 4 °C. The cells were washed and analyzed by multicolor flow cytometry with the Gallios Flow Cytometer (Beckman Coulter Inc.).

### Statistical analyses

Graph pad prism (Version 5.04) was applied for statistical analysis with the Mann-Whitney U test. Results were considered statistically significant for *P*<0.05 (*) or (#) and highly significant for *P*<0.01 (**) or (##).

## Abbreviations

AnxA5: AnnexinA5
APC: antigen-presenting cell
ATP: adenosine triphosphate
CFSE: carboxyfluorescein succinimidyl ester
CT: chemotherapy
CTL: cytotoxic T lymphocyte
DAMP: damage-associated molecular patterns
DC: dendritic cell
DTIC: dacarbazine
ELISA: enzyme-linked immunosorbent assay
FITC: fluorescein isothiocyanate
GM-CSF: granulocyte macrophage colony-stimulating factor
Gy: gray
HMGB1: high mobility group protein B1
HT: hyperthermia
MDSC: myeloid derived suppressor cell
MLKL: mixed lineage kinase domain-like
MyD88: myeloid differentiation primary response gene (88)
NK cells: natural killer cells
NLRP3: NOD-like receptor family, pyrin domain containing 3
PI: propidium iodide
RAG: recombination activating proteins
RIP: receptor-interacting protein
RCT: radiochemotherapy
RT: radiotherapy
SN: supernatant
TLR: toll like receptor
Treg: regulatory T cell
w/o: without
U: units
X-ray: ionizing irradiation
zVAD-fmk: carbobenzoxy-valyl-alanyl-aspartyl-[Omethyl]-fluoromethyl-ketone
