# Supplementary figures and images for: Modulation of radiochemoimmunotherapy-induced B16 melanoma cell death by the pan-caspase inhibitor zVAD-fmk induces anti-tumor immunity in a HMGB1-, nucleotide- and T-cell-dependent manner
Source: Cell Death Dis. 2015 May 14;6(5):e1761–. doi: 10.1038/cddis.2015.129 (PMC4669707; doi:10.1038/cddis.2015.129)

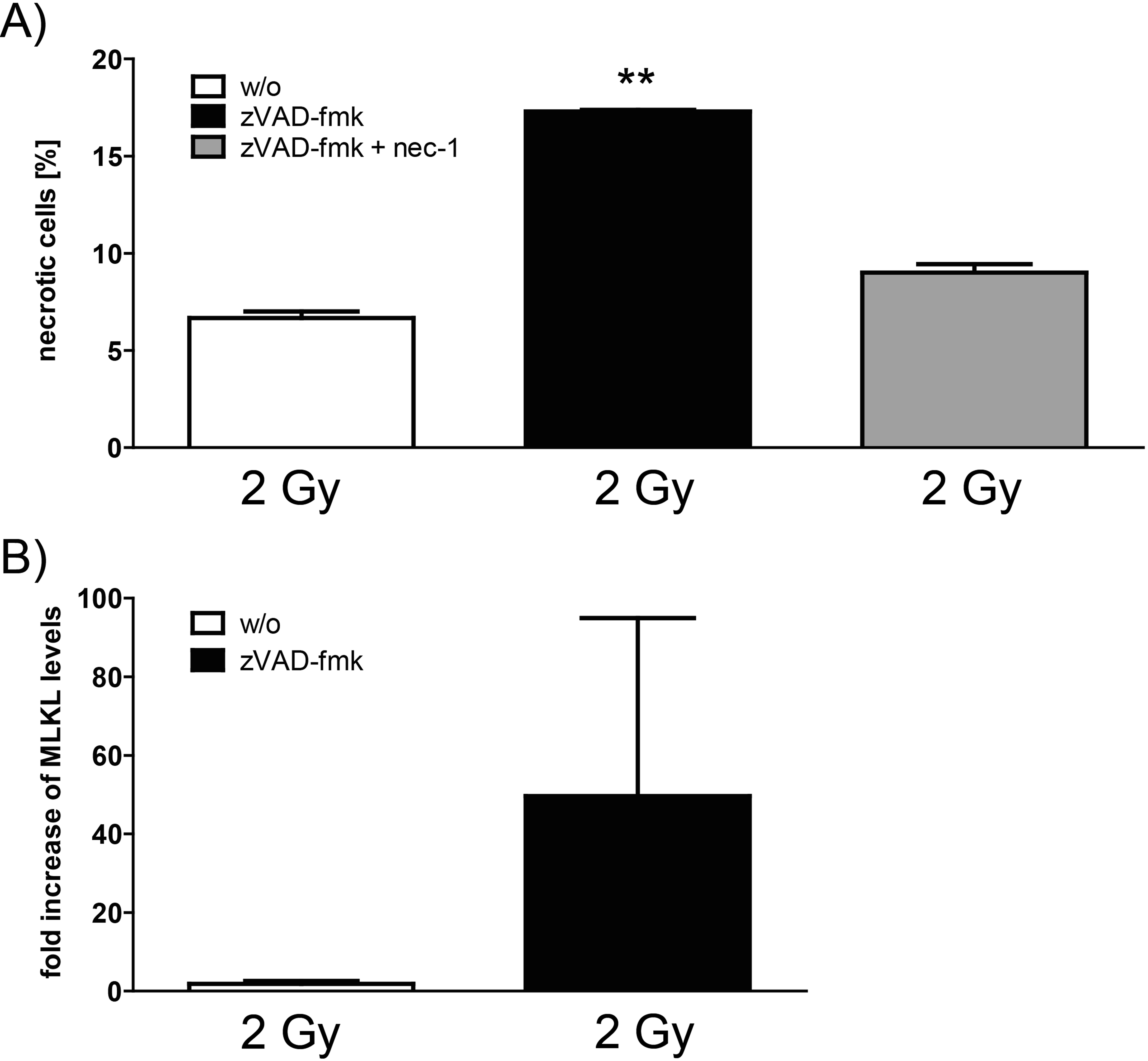

Supplement: Supplementary Figure 1 [file cddis2015129x1.tif]

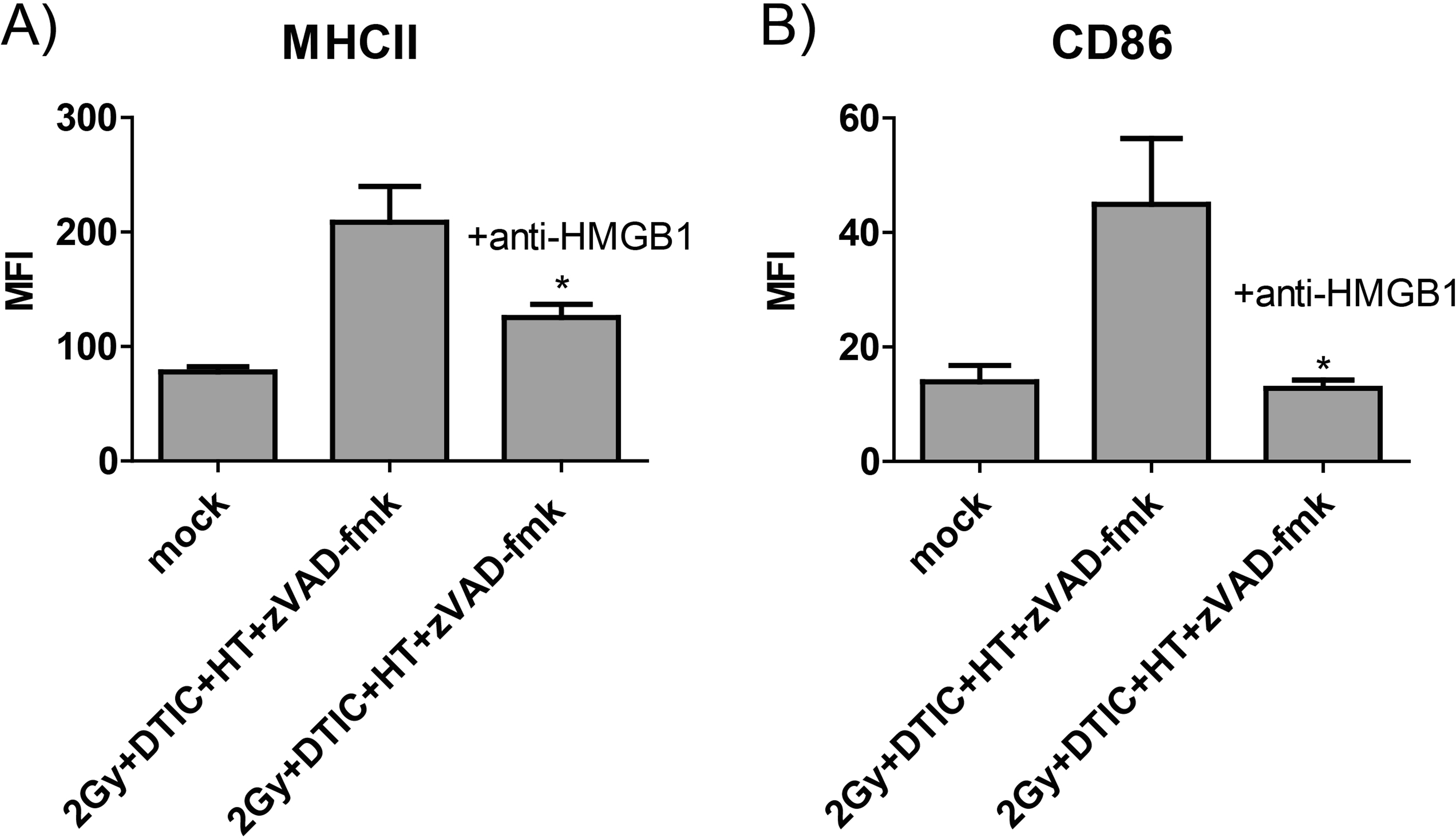

Supplement: Supplementary Figure 2 [file cddis2015129x2.tif]

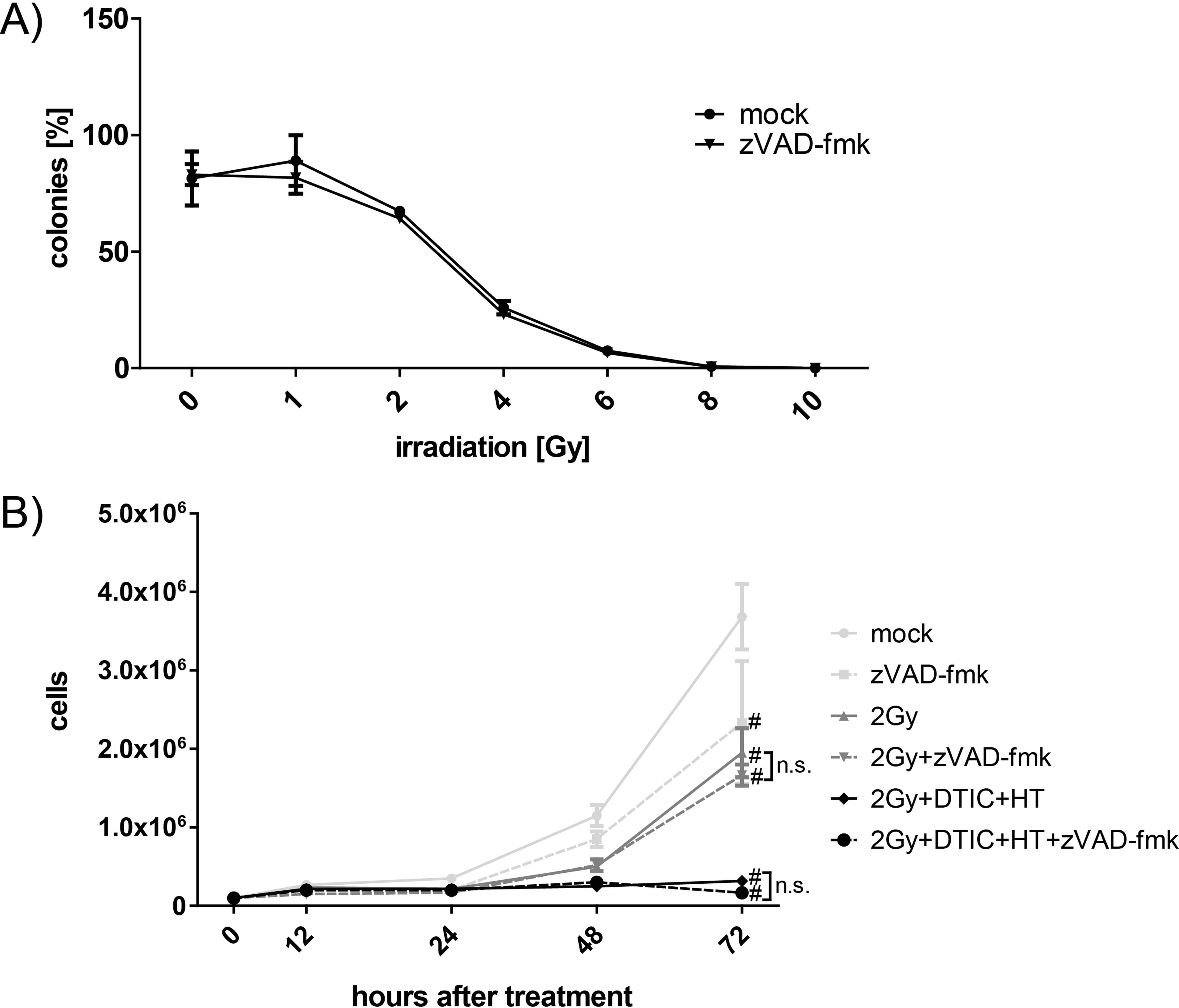

Supplement: Supplementary Figure 3 [file cddis2015129x3.tif]

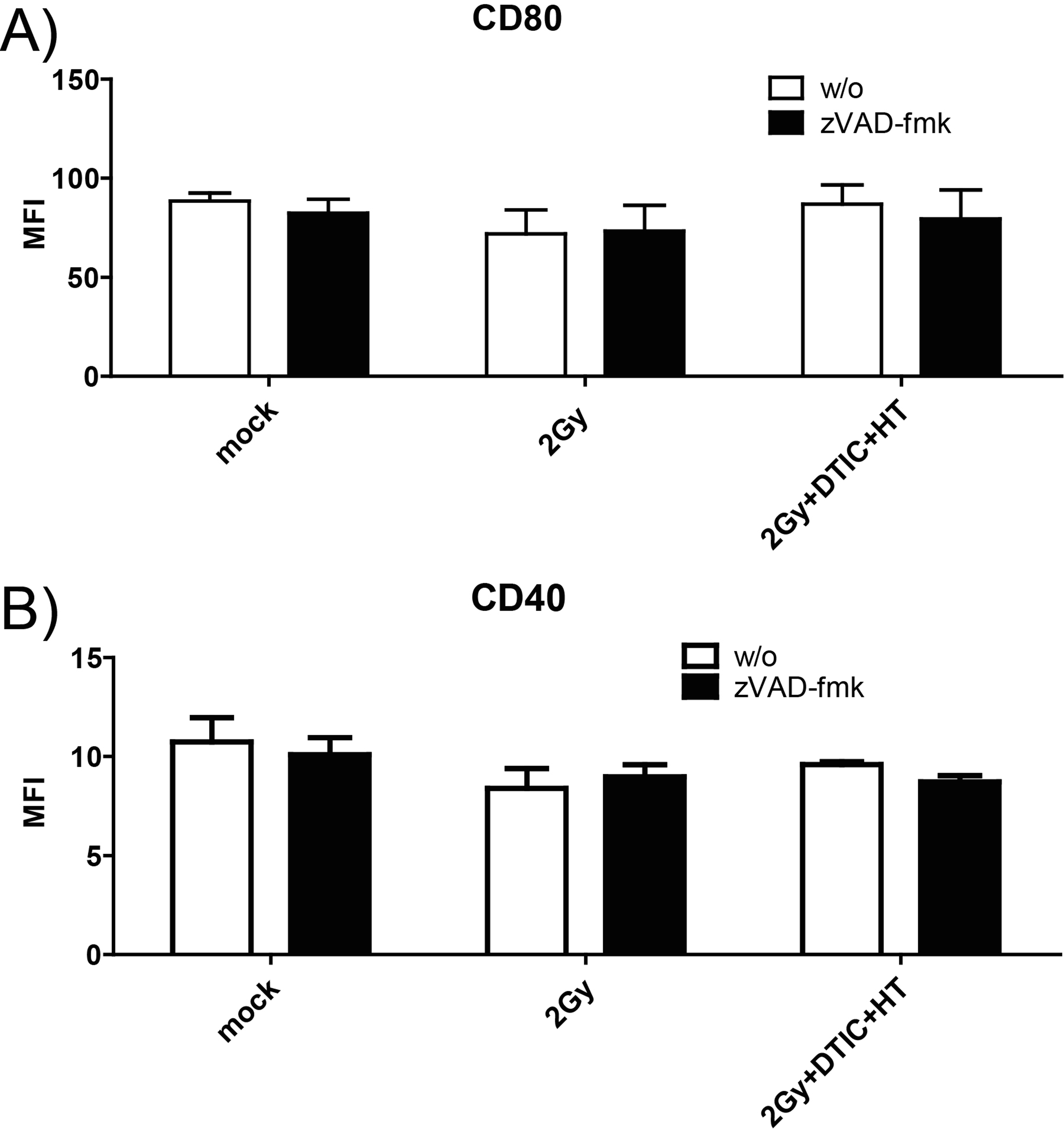

Supplement: Supplementary Figure 4 [file cddis2015129x4.tif]
