# Supplementary material for: Modulation of radiochemoimmunotherapy-induced B16 melanoma cell death by the pan-caspase inhibitor zVAD-fmk induces anti-tumor immunity in a HMGB1-, nucleotide- and T-cell-dependent manner
Source: Cell Death Dis. 2015 May 14;6(5):e1761–. doi: 10.1038/cddis.2015.129 (PMC4669707; doi:10.1038/cddis.2015.129)
Supplement: Supplementary Figure Legends [file cddis2015129x5.doc]

**Supplementary Legends to the Figures**

**Suppl. Figure 1: zVAD-fmk-induced necrosis and MLKL mRNA expression of irradiated melanoma cells.**

Necrosis of B16 mouse melanoma cells was analyzed with two color flow cytometry by staining the cells with AnxA5-FITC and PI 24h after irradiation with 2Gy in the presence or absence of zVAD-fmk or zVAD-fmk plus nec-1 (A). The expression of mRNA of MLKL was analyzed at the same time point with q-RT-PCR (B). Representative data of one out of three experiments, each performed in duplicates, are presented as mean ± S.D. ** P < 0.01; nec-1: necrostatin-1; MLKL: mixed lineage kinase domain-like.

**Suppl. Figure 2: Impact of anti-HMGB1 antibody on the surface expression of activation markers on DCs after contact with supernatants of treated melanoma cells in the presence of zVAD-fmk.**

The expression of the activation markers MHCII (A) and CD86 (B) on the surface of bone marrow derived dendritic cells (DCs) of C57/BL6 wild type mice was analyzed by multicolor flow cytometry after contact with supernatants (SNs) of B16 mouse melanoma cells obtained 24h after the treatments. The tumor cells were treated with ionizing irradiation with 2Gy in combination with the chemotherapeutic agent dacarbazine (DTIC; 250 µM), hyperthermia (HT; 41.5°C for 1 hour), and the pan-caspase inhibitor zVAD-fmk (50 µM) in the absence or presence of a neutralizing antibody against HMGB1 (A, B). Representative data of one out of 3 experiments, each performed in duplicates, are presented as mean ± S.D. ** P < 0.01 related to treated tumor cells in the absence of anti-HMGB1 antibody; MFI: mean fluorescence intensity; mock: SN of untreated tumor cells.

**Suppl. Figure 3: Impact of zVAD-fmk on colony formation and growth of melanoma cells.**

Colony formation (A) or growth (B) of B16 mouse melanoma cells was determined by counting colonies (A) or single cells (B) after the respective treatments with irradiation or irradiation with 2Gy in combination with the chemotherapeutic agent dacarbazine (DTIC; 250 µM) and hyperthermia (HT; 41.5°C for 1 hour), in the absence or presence of zVAD-fmk. Data of three experiments, each performed in duplicates, are presented as mean ± S.D. ^#^ P < 0.05; ^##^ P < 0.01 related to untreated tumors (mock); n.s.: not significant.

**Suppl. Figure 4: Surface expression of CD80 and CD40 on DCs after contact with supernatants of treated melanoma cells in the presence or absence of zVAD-fmk.** The expression of the activation markers CD80 (A) and CD40 (B) on the surface of bone marrow derived dendritic cells (DCs) of C57/BL6 wild type mice was analyzed by multicolor flow cytometry after contact with supernatants (SNs) of B16 mouse melanoma cells obtained 24h after the respective treatments. The tumor cells were treated with ionizing irradiation with 2Gy alone or in combination with the chemotherapeutic agent dacarbazine (DTIC; 250 µM) and hyperthermia (HT; 41.5°C for 1 hour), in each case in the absence (w/o) or presence of the pan-caspase inhibitor zVAD-fmk (50 µM). Data of three experiments, each performed in triplicates, are presented as mean ± S.D. ** P < 0.01 related to samples without (w/o) inhibitor; MFI: mean fluorescence intensity; mock: SNs of untreated tumor cells.
